# Substantiating Clinical Effectiveness and Potential Barriers to the Widespread Implementation of Spinal Cord Injury Telerehabilitation: A Systematic Review and Qualitative Synthesis of Randomized Trials in the Recent Past Decade

**DOI:** 10.1089/tmr.2020.0026

**Published:** 2021-02-24

**Authors:** Seungbok Lee, Jeonghyun Kim, Jongbae Kim

**Affiliations:** ^1^ Yonsei Enabling Science Technology and Research Clinic, Yonsei University, Wonju, Republic of Korea.; ^2^ Department of Physical Medicine and Rehabilitation, Seoul National University College of Medicine, Seoul, Republic of Korea.; ^3^ Department of Occupational Therapy, Yonsei University College of Health Sciences, Wonju, Republic of Korea.

**Keywords:** SCI, spinal cord injury, technology, telemedicine, telerehabilitation

## Abstract

**Introduction:** Telemedicine across many specialties in clinical practice has been established in the literature regarding technology platforms, privacy issues, cost, and clinical effectiveness. However, the lack of data in these areas applicable to spinal cord injury telerehabilitation (teleSCI) still exists. The gaps in these knowledge areas continue to hinder its widespread implementation and serve as pathways for focused efforts in teleSCI research.

**Objective:** This systematic review aims to substantiate the clinical effectiveness and potential barriers to teleSCI implementation by verifying the statistical significance of various clinical outcomes from randomized trials published within the recent past decade.

**Methods:** A qualitative synthesis of randomized studies, conducted across various regions, was systematically reviewed after identifying relevant records from database search engines. Applied filters in the search included publication dates (2010–2020), humans, full-text, and no language preference. The 13 studies were selected per Preferred Reporting Items for Systematic Reviews and Meta-Analyses flow diagram, and the risk of bias across studies was evaluated by using the Physiotherapy Evidence Database scale of quality assessment.

**Results:** Quantitative outcome measurements demonstrated positive impact across studies: 79.1% (34/43) of all measurements were statistically significant for positive outcomes and 18.6% (8/43) yielded no effect but were significant. Primary outcomes addressed various spinal cord injury (SCI) management areas; 38.5% (5/13) of studies also assessed secondary outcomes. Interventional platforms were conventional technologies used in telemedicine. One study (7.7%) achieved data encryption; no studies presented cost-analysis data.

**Conclusion:** The majority of studies demonstrated significant positive outcomes to validate teleSCI clinical effectiveness through conventional technology. These results further expand our understanding of teleSCI's impact and its demonstrated potential for improving SCI individuals' lives. However, heterogeneity of selected studies limits the conclusive recommendations to address potential barriers to its widespread implementation. Moreover, the development of new data is warranted to promote “buy-in” of widespread teleSCI implementation.

## Introduction

Spinal cord injury or dysfunction (SCI/D) can result from either traumatic or nontraumatic occurrences and renders life-altering events. Its clinical manifestations involve changes in underlying strength, sensation, and other bodily function that causes paralysis and disability.^1^ Other consequences manifest in varying degrees with profoundly compromised function in necessary life activities.^2^ Furthermore, secondary medical complications associated with multiorgan systems including neurogenic bowel, lower urinary tract disorder, pressure ulcers, endocrine and metabolic abnormalities, and cardiopulmonary and hemodynamic instability along with sexual dysfunction commonly occur after discharge from acute inpatient rehabilitation.^3^ These comorbidities can lead to unfortunate losses physically, emotionally, socially, and economically.^4^

Secondary complications require costly and time-consuming medical management by qualified specialty care providers in a clinical setting. The morbidities yield severely compromised levels of activity and participation, ultimately impacting the quality of life (QoL) for many, with death resulting in severe cases.^5^ The situation can be devastating not only for patients but also for caregivers and clinical providers involved in their care. Therefore, it is essential to prevent and systematically manage these complications. However, continuous and appropriate health services for SCI/D individuals are denied under many circumstances. Many of these individuals cannot access specialty clinics due to barriers in economic burdens, transportation, and remote locations.^6^

In the efforts to combat such barriers, the advent of telemedicine has propelled ways for the delivery of medical care from remote locations through information telecommunication technology (ICT).^7^ The evolving field of telemedicine, in past decades, has revealed innovative options in telerehabilitation to address underlying challenges in remotely managing patients requiring rehabilitation. In 2016, the concept of spinal cord injury telerehabilitation (teleSCI) was officially introduced at the International Spinal Cord Society Annual Scientific Meeting in Vienna, Austria, by an international panel of leaders through an instructional telemedicine course.^8^ Furthermore, evidence in the literature shows the efficacious role of telemedicine within other clinical specialties—internal medicine and mental health—with high levels of satisfaction among patients who reside in remote locations.^9^

Telerehabilitation in spinal cord injury (SCI) management has been applied and reported to have had successful outcomes in some global areas.^10^ It is especially beneficial for those who are disadvantaged by barriers to accessing specialty care clinics. From the perspective of “taking ownership,” Kryger et al. validate that appropriate use of technology enhances empowerment to participants promoting a sense of responsibility in being more involved and better educated about their care and overall well-being.^11^ However, mobile and telehealth interventions appear to be used less commonly in teleSCI management.^12,13^

Furthermore, there has been insufficient evidence generated, even in the recent past decade, which validates its clinical efficacy in SCI individuals. The lack of data is particularly evident throughout many Asian countries, presumably influenced by economic, political, and cultural factors.^10^ In the Republic of Korea (S. Korea), debates regarding the feasibility of teleSCI among health care experts are ongoing, whereas uncertainties in cost-effectiveness leave doubts in physicians' minds regarding widespread implementation across medical specialties.^14^

Health care experts still consider teleSCI to be in its developing stages, which require fine tuning in many aspects.^15^ The associated privacy concerns present as deterrence for policymakers. The data regarding cost–benefits are insufficient to convince health economic experts, whereas sparse evidence regarding clinical efficacy and reimbursement inevitably fails to reach a consensus among physicians.^16–18^ These are some of the demanding challenges remaining to be addressed and require solutions to overcome the lingering concerns toward telemedicine implementation. Gaps in these knowledge areas continue to maintain uncertainties in teleSCI clinical practice and research. This systematic review aims to substantiate the clinical effectiveness of teleSCI and confirm existing potential barriers in its widespread global implementation by verifying the statistical significance of clinical outcomes from randomized trials published within the recent past decade.

## Methods

### Database search

**FIG. 1.**
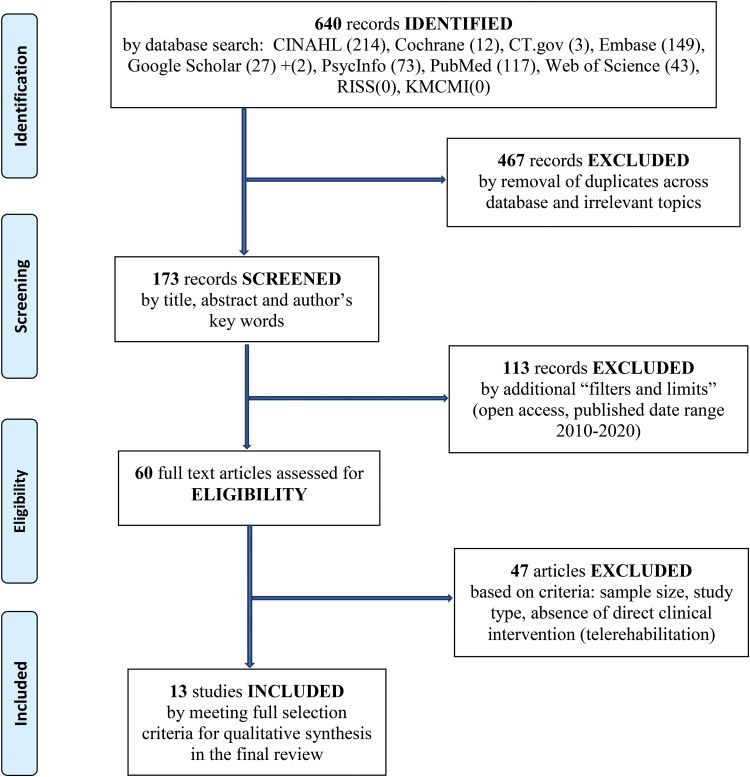
Preferred Reporting Items for Systematic Reviews and Meta-Analyses flowchart: selection of studies by database literature search.

The first author performed the initial search using key search terms and appropriate Boolean phrases: telerehabilitation OR telemedicine AND “spinal cord injury” OR “SCI.” Search engines included CINAHL (*n* = 214), PubMed (*n* = 117), and Clinicaltrials.gov (*n* = 3). A research assistant independently performed a search (March 15, 2020) on two Korean databases: Research Information Sharing Service and Korea Medical Citation Medical Index. However, these did not yield any relevant articles based on our search criteria. Our salient efforts involved a comprehensive search of the gray literature using Web of Science (*n* = 43), EMBASE (*n* = 149), Cochrane Library (*n* = 12), PsyINFO (*n* = 73), and Google Scholar (*n* = 29). We assessed a total of 640 records identified from 10 search engines aforementioned to narrow the selection field. The following filters were applied: (1) publication date range from January 1, 2010 to January 1, 2020, (2) human subjects, (3) open access with full-text articles, and (4) no language preference specified. The flow diagram in Figure 1 shows the summary of database research.

### Screening and eligibility criteria

The identified records were assessed by research assistants, giving attention to the title, abstract contents, and key phrases per our selection criteria. This review focused uniquely on randomized trials, including feasibility and pilot studies with respect to the study design. Our criteria for inclusion and exclusion are outlined in detail.

#### Inclusion

a. Randomized controlled or clinical trials (RCTs) including pilot and feasibility studies.
b. Study participants with underlying SCI/D (spinal cord injury or dysfunction).
c. Study participants are adult males or females of 18 years or greater.
d. Sample size greater than eight participants per study.
e. Health care and related services delivered through teleSCI platform with outcomes assessment in SCI/D management.
f. Intervention delivered using technology from home or community setting with interactions between participants and clinical providers (physicians, nurses, allied health professionals, and licensed or qualified rehabilitation counselors).

### Exclusion

We excluded case studies, case series presentations, observational studies, articles with fewer than eight study participants, and all review types of studies. Study protocols merely proposing a study design without resultant data or interventional outcomes were all excluded. Since telerehabilitation is still considered evolving and require fine tuning,^19^ particularly in technology advancements, our review focused on current research spanning the past decade. Studies published outside of the specified date range—between 2010 and 2020—were also excluded. After eliminating duplicates and other irrelevant articles, 13 studies met our criteria and were included in this review.

### Data collection process

The first author performed data extraction from 13 selected articles. At the time of data acquisition, one of the designated coauthors withdrew and was pre-empted from further collaboration. Subsequently, any discrepancies were resolved by consensus among the three coauthors.

### Extracted data information

The computing system in the research laboratory served as the central management system for acquired data storage. Record keeping of all information was maintained separately on an excel spreadsheet. Addendum notes were also generated for organizing or information and recorded using a word document. The items of extracted data were categorized as follows: characteristics of (1) selected studies, (2) study population, (3) study methods, and (4) study results with outcome data. The summary of these data categories is given in Table 1.

**Table 1.** Summary of Data Extracted

Data categories	
Selected studies	Author, title, institution, country of origin, objectives, digital object identifier, first date of article submission and final publication dates
Study population	Sample size, age, gender, SCI level or type (ASIA scale classification), comorbidities
Study methods	Design, setting, geographic location, data collection method and tools, process of recruitment, randomization and allocation concealment, follow-up period, interventional platform, secondary technology, and data security
Results and data outcome	Primary and secondary outcomes, observed outcome measurements, intervention results and statistics analysis, adverse events, and attrition rate

ASIA, American Spinal Injury Association; SCI, spinal cord injury.

### Information for assessment of the risk of bias

The quality of the selected studies was evaluated by assessing the risk of bias, using the quality checklist assessment formulated by Physiotherapy Evidence Database (PEDro) scale in reporting data.^20^ In systematic reviews, evaluating the quality of RCTs is common practice. However, the reliability of data obtained with most quality assessment scales has not been established.^21^ Maher et al.^21^ described in their report of two studies designed to investigate the reliability of data obtained with the PEDro scale developed to rate RCTs' quality evaluating physical therapist interventions. The scale is a valid measure of the methodological quality of clinical trials.

## Results

### Selection and characteristics of studies

The Preferred Reporting Items for Systematic Reviews and Meta-Analyses flow diagram shown in Figure 1 summarizes the systematic approach to our record search. Identified records were assessed based on our selection criteria. Through a stepwise process of screening and exclusion, 13 total studies met our criteria. The characteristics of the selected studies included in the final review are summarized in Table 2.

**Table 2.** Summary of the Characteristics of Selected Studies

Author	Title	Title	Journal	Design	Sample	Population	Intervention technology	Follow-up
Hossain et al.^6^	A pilot randomized trial of community-based care following discharge from hospital with a recent spinal cord injury in Bangladesh	May, 2016	Clinical Rehabilitation	Randomized trial (pilot)	*N* = 30	SCI	“Care Pack” = Telephone contacts and home-visits	24 months
Houlihan et al.^32^	A pilot study of a telehealth intervention for persons with spinal cord dysfunction	June, 2013	Spinal Cord	RCT	*N* = 142	SCI, MS	“Care Call” = automated interactive voice response system	6 months
Migliorini et al.^22^	A randomized control trial of an internet-based cognitive behavior treatment for mood disorder in adults with chronic spinal cord injury	December, 2015	Spinal Cord	RCT (prospective parallel waitlist)	*N* = 48	SCI	Telephone-interview: ePACT	15 months
Arora et al.^23^	Cost-effectiveness analysis of telephone-based support for the management of pressure ulcers in people with spinal cord injury in India and Bangladesh	August, 2017	Spinal Cord	RCT	*N* = 115	SCI	Telephone-based support	12 weeks
Kowalczewski et al.^24^	In-home telerehabilitation improves tetraplegic hand function	March, 2011	Neurorehabilitation and Neural Repair	RCT (cross-over)	*N* = 13	SCI	Tele-supervised exercise therapy	9 months
Hearn and Finlay^25^	Internet-delivered mindfulness for people with depression and chronic pain following spinal cord injury: a randomized, controlled feasibility trial	March, 2018	Spinal Cord	Randomized trial (feasibility)	*N* = 67	SCI	Web-based online mindfulness training	8 weeks
Houlihan et al.^26^	Randomized trial of a peer-led, telephone-based empowerment intervention for persons with chronic spinal cord injury improves health self-management	March, 2017	Archives of PM&R	RCT	*N* = 84	SCI	Telephone-based, text and e-mail support by peer-health coaches	6 months
Kryger et al.^11^	The effect of the interactive mobile health and rehabilitation system on health and psychosocial outcomes in spinal cord injury: a RCT	August, 2019	Journal of Medical Internet Research	RCT	*N* = 38	SCI	Smart-device interactive mobile application modules and web-based portal monitoring	9 months
Coulter et al.^27^	The effectiveness and satisfaction of web-based physiotherapy in people with spinal cord injury: a pilot RCT	2017	Spinal Cord	RCT (pilot)	*N* = 24	SCI	Web-based PT exercise program with telephone interview	8 weeks
Worobey et al.^28^	Investigating the efficacy of web-based transfer training on independent wheelchair transfers through RCTs	2018	Archives of PM&R	RCT	*N* = 71	SCI, MS, amputees, others	Web-based training of wheelchair transfers	1–2 days postintervention
Rimmer et al.^29^	Telehealth weight management intervention for adults with physical disabilities a RCT	December, 2013	American Journal of PM&R	RCT	*N* = 102	SCI, MS, CP, spina bifida, CVA, SLE	Web-based remote coaching and telephone support	9 months
Dorstyn et al.^30^	Work and SCI: a pilot randomized controlled study of an online resource for job-seekers with spinal cord dysfunction	September, 2018	Spinal Cord	RCT (pilot)	*N* = 48	SCI/D	Web-based online informational intervention	4 weeks
Shen et al.^31^	Clinical treatment of orthostatic hypotension after spinal cord injury with standing training coupled with a remote monitoring system	December, 2014	Medical Science Monitor	RCT	*N* = 36	SCI/D	Remote monitoring of electric bed uprise training with wearable remote wireless multi-parameter dynamic monitoring system	30 days

CP, cerebral palsy; CVA, cerebrovascular accident; ePACT, electronic personal admin of cognitive therapy; MS, multiple sclerosis; PT, physical therapy; RCT, randomized controlled or clinical trial; SCI/D, spinal cord injury or dysfunction; SLE, systemic lupus erythematosus.

### Risk of bias across studies

The risk of bias was assessed across studies using the PEDro scale. Nine studies scored 9 or greater out of 11, whereas three studies scored 7 out of 11, and one study scored 6 out of 11. Potential bias across studies appears to be associated with blinding and allocation concealment. Quality assessment based on the PEDro scale revealed that 38.5% (5/13) of selected studies achieved allocation concealment. Blinding of participants, clinicians administering interventions, and assessors measuring outcome were reported as 46.2% (6/13), 15.4% (2/13), and 46.2% (6/13) across studies, respectively. Table 3 gives a summary of the assessment.

**Table 3.** Summary of the Characteristics of Selected Studies, Primary Outcomes and Measurements

Author	Intervention setting	Geographic location	Data encryption	PEDro scale risk of bias	Attrition rate	Primary outcomes	Outcomes measurement
Hossain et al.^6^	Home, community	Bangladesh	None reported	8/11	6.7%	All cause mortality	Two participants died; mortality rate of 7% (95% CI: 2 to 21)
Houlihan et al.^32^	Community	United States	None reported	8/11	6.3%	Reduced presence of pressure ulcers at 6 months in women (*p* < 0.0001); reduced 6-month severity of depression (age and gender adjusted; *p* < 0.047)	No significant impact on health-care utilization (OR = 1.8, *p* = 0.07)
Migliorini et al.^22^	Home	Australia	None reported	8/11	22.9%	Mood improvement with life satisfaction	Within-group analyses showed significant mood improvement (ES = 0.4), anxiety (ES = 0.4), stress (ES = 0.3) and higher life-satisfaction (ES = 0.2)
Arora et al.^23^	Tertiary care center	Bangladesh, India	None reported	9/11	Not reported	Cost–benefit and health outcomes (ulcer size reduction and QALY gained	Between-group difference for mean reduced pressure ulcer size (95% CI: −3.12 to −4.32), corresponding QALYs = 0.027 (95% CI: 0.004 to 0.051), 87% cost-effectiveness by sensitivity analyses
Kowalczewski et al.^24^	Home	Australia, Canada	None reported	10/11	All participants completed study	ARAT	Arm and hand strengths' score (*p* < 0.01) improved after ET
Hearn and Finlay^25^	Home, community	United Kingdom	Data encrypted	8/11	35.8%	Depression symptom severity	Depression significantly reduced more by mindfulness than psychoeducation (mean_diff = −1.50; 95% CI: −2.43 to −0.58)
Houlihan et al.^26^	Home, community	Canada, United States	None reported	8/11	9.9%	Health self-management	Peer-led health self-management yielded significant change in PAM scores (*p* = 0.047)
Kryger et al.^11^	Home	United States	None reported	9/11	13.2%	Health outcomes = UTI, pressure sores, emergency room visits, hospital admissions	Intervention significantly reduced UTIs (*p* = 0.03) with nonsignificant psychosocial outcomes trending toward reduced mood symptoms
Coulter et al.^27^	Home, community	United Kingdom	None reported	7/11	12.5%	6 MPT or 6 MWT depending on mobility	Between-group differences were not significant but more pronounced for 6-MWT
Worobey et al.^28^	Home	United States	None reported	8/11	7%	Transfer techniques @ baseline, skills-acquisition immediate post-training, and skills-retention post 1–2 days follow-up	Web-based transfer training showed improvement (*p* = 0.05)
Rimmer et al.^29^	Home	United States	None reported	7/11	10.8%	Biomed = weight, body mass index; barriers to activity; activity = aerobic exercise; strength exercise; total-exercise; nutrition = fat score; fiber score; fruit/veggie score	Bodyweight difference between groups was significant in group and time interaction on statistic analysis (*p* < 0.01)
Dorstyn et al.^30^	Home, community	Australia	None reported	7/11	36%	25-item JSES	High uptake of work and SCI resources through learning module; high attrition rate observed with intention-to-treat analyses failing to reach statistical significance
Shen et al.^31^	Home	China	None reported	6/11	11.1%	Responses to electric uprise bed training and compare training efficiency	Tilt-table training improved in systolic and diastolic orthostatic blood pressure changes

ARAT, Action Research Arm Test; CI, confidence interval; ES, effect size; ET, exercise therapy; JSES, Job Procurement Self-Efficacy Scale; MPT, Min Push Test; MWT, Min Walk Test; OR, odds ratio; PAM, Patient Activation Measure; PEDro, Physiotherapy Evidence Database; QALY, quality-adjusted life years; UTI, urinary tract infection.

### Synthesis of results

#### Selected studies

The following is a summary of the characteristics of the selected studies included in this review.^6,11,22–31^ The affiliated institution, country of origin, study title, study objectives, journals in which they were published, along with digital object identifier numbers and date of articles' first submission were extracted. After agreement by coauthors, studies were included, following independent search and screening efforts without the assistance of a librarian. Our criteria for publication date ranged between 2010 and 2020 since a decade was believed to be an extended period for advancements in IT device technology for their potential application in telemedicine. The earliest publication among selected studies was from 2011, whereas the most recent was from 2019.

#### Study population

The total number of participants across studies was 818 and ranged from 13 to 142, with a mean of 62.9 participants.^32,24^ The mean of male participants across all studies was 62.96% (*n* = 515/818). Three of the studies included >50% female participants in both experimental and control groups,^24^ and two studies had >71% in female participants.^27,28^ Age was reported in years by mean with standard deviation, among seven studies according to intervention (range 29–51.5) and control (range 38–52.8) groups.^6,11,22,23,27,28,31^

Ten studies focused strictly on SCI, whereas one study included multiple sclerosis (MS).^23^ One study included MS and amputees,^28^ whereas another study included MS, cerebral palsy (CP), stroke, spina bifida, and lupus.^29^ Two studies reported on the American Spinal Injury Association (ASIA) scale classification with SCI type by specific neurological levels of injury.^21,25^ Majority did not use ASIA scale classification, whereas some labeled participants as “tetraplegia” versus “paraplegia” without detailed neurological levels.^11,22,24,27,28,30,31^ Six studies reported completeness or incompleteness of injury.^11,22,24,27,30,31^ One study that included MS participants reported only neurological levels of injury for its SCI participants.^32^ Two studies, which included disabilities of varying disorders (MS, CP, stroke, amputees, and lupus), did not specify neurological levels of injury nor ASIA classification.^28,29^ One study did not report types or levels of injury.^23^

Comorbidities of participants reported by seven studies included pressure ulcers,^11,23,32^ depression,^11,22,32^ pain,^25^ diabetes,^26^ and cardiovascular abnormalities.^26,31^ The remaining six studies did not report comorbidities of participants.^6,24,27–30^

#### Study methods

The settings for conducting the intervention were in the participants' home for all studies (some studies indicated “community” as a designated remote location and that was considered as “home”). In contrast, one study was conducted in a laboratory-simulated home setting.^23^ Geographically, studies were conducted in various countries including the United States,^26,27–29,32^ Canada,^24,26^ Australia,^22,24,30^ United Kingdom,^25,27^ Bangladesh,^21^ India,^23^ and China.^31^

Various methods of data collection were reported, including telephone interviews and/or in combination with written questionnaires.^6,21–23,25,26,27–30,32^ One study added home visits with telephone interviews.^6^ Other studies used clinical assessment,^23,24,31^ survey forms,^29^ and web-portal with software applications.^11^ Most studies used one or more standardized tools for data collection. Among these, various tools were used to assess the QoL^6,11,23,25,30^: WHO-QOL-BREF, HR-QoL, PWI, SCL-EWL, PHQ-9, QALY, and PUSH. Pain, depression, and anxiety symptoms were assessed by respective authors in the studies using NRS, PCS, CES Depression Scale, DASS-21, BDI-II, and HADS. Levels of disability, activity, and independence were measured by CHART-SF, PADS, SCI-SC scale, SCIM-III, COPM, WHODAS, and CHART-SF. Mindfulness of self-management in health was also measured using FFMQ, PAM, PACIC, ASIS, and CHIEF-SF. Other tools used in studies included ARAT and RAHFT to measure hand function and strength, whereas TAI scores evaluated the performance of wheelchair transfer techniques.^24^ Self-reported measures for seeking employment were measured using JSES and LOT-R.^29^ Refer to Appendix A1 for abbreviations.

All studies in this review are RCTs. Four of these are pilot or feasibility studies.^6,25,27,30^ Six studies reported in detail on the process of recruitment, randomization, and ascertaining of allocation concealment.^6,22,26,27,28,30^ Five reported on only the recruitment process.^23–25,27,29^ Two studies neglected to provide any information regarding these processes in their study design.^25,32^ The quality assessment across studies (Table 4) revealed that six studies achieved blinding of all study participants.^6,22–26^ Two studies secured blinding among those who administered intervention.^11,24^ Blinding of those who assessed at least one key outcome was attained in 6 of the 13 studies.^6,11,23–25,29^

**Table 4.** Physiotherapy Evidence Database Scale for Assessing Quality: Risk of Bias in Studies

	Hossain et al.^6^	Houlihan et al.^32^	Migliorini et al.^22^	Arora et al.^23^	Kowalczewski et al.^24^	Hearn and Finlay^25^	Houlihan et al.^26^
1. Eligibility criteria were specified	p.782, Methods	Table 1	p.695, Methods	p.1072, Participants	p.413, Participants	p.751, Participants	p.1068, Participants
2. Subjects were randomly allocated to groups	p.782, Methods	p.716, Participants	p.696, Methods	p.1072, Intervention	p.413, Participants	p.752, Procedure	p.1068, Trial design
3. Allocation was concealed	p.782, Methods	N	p.695, Methods	p.3, Figure 1{Arora&Harve}	N	N	N
4. Groups were similar at baseline regarding the most important prognostic indicators	Table 1	Table 3	Table 1	Table 1	Table 1	Table 2	Table 2
5. There was blinding of all subjects	p.782, Methods	Abstract	p.696, Methods	N	p.413, Design	p.752, Procedure	p.1068, Trial design
6. There was blinding of all therapists who administered the therapy	N	N	N	N	p.415, Primary outcome	N	N
7. There was blinding of all assessors who measured at least one key outcome	p.783, Methods	N	N	p.3, Assignment	p.415, Primary outcome	p.752, Procedure	N
8. Measures of at least one key outcome were obtained from >85% of the subjects initially allocated to groups	Table 2	Table 4, Figures 2a, 2b	N	Tables 3, 4	p.413, Table 2 and Participants	N {p.755, Compliance}	p.1071, Results
9. All subjects for whom outcome measures were available received the treatment or control condition as allocated or, where this was not the case, data for at least one key outcome were analyzed by “intention to treat”	Table 2, Figure 1	p.717, Stats analysis	p.697, Analyses	Tables 3, 4	Table 2	p.754, Results	p.1071, Engagement
10. Results of between-group statistical comparisons are reported for at least one key outcome	N	p.717, Results	Table 2	Table 5	p.417, Comparison	Table 3	Table 4
11. The study provides both point measures and measures of variability for at least one key outcome	N	Figures 2a, 2b	Tables 3, 4	Table 5	p.417, Effect size	p.756, Effects	Table 4
Total	8/11	8/11	8/11	9/11	10/11	8/11	8/11
Information in each cell indicates the corresponding “page number” or “section” (Abstract, Methods, Results, Participants, Design, Statistics analysis, Outcomes, Comparison, Effect size, Tables, Figures, etc.) under which each of the 11 areas of quality assessment was reported by respective authors in the original article.							
*N*, none reported.							

	Kryger et al.^11^	Coulter et al.^27^	Worobey et al.^28^	Rimmer et al.^29^	Dorstyn et al.^30^	Shen et al.^31^	
1. Eligibility criteria were specified	p.3, Recruitment	p.384, Methods	p.10, Participants	p.1085, Participants	p.222, Eligibility	p.2768, Subjects	
2. Subjects were randomly allocated to groups	p.3, Design	p.384, Methods	p.10, Participants	p.1086, Recruitment	p.223, Procedure	p.2768, Subjects	
3. Allocation was concealed	N	N	p.10, Participants	N	p.223, Procedure	N	
4. Groups were similar at baseline regarding the most important prognostic indicators	Table 1	Table 1	Table 1	Table 2	Table 1	Table 1	
5. There was blinding of all subjects	N	N	N	N	N	N	
6. There was blinding of all therapists who administered the therapy	p.3, Design	N	N	N	N	N	
7. There was blinding of all assessors who measured at least one key outcome	p.3, Design	N	N	p.1087, Measures	N	N	
8. Measures of at least one key outcome were obtained from more than 85% of the subjects initially allocated to groups	p.6, Results, Figure 2	p.384, Results	p.13, TAI, Table 1	N	N	N	
9. All subjects for whom outcome measures were available received the treatment or control condition as allocated or, where this was not the case, data for at least one key outcome were analyzed by “intention to treat”	Table 3	Table 2	Table 1	p.1088, Stats analysis	p.225, Preliminary effects	Table 5	
10. Results of between-group statistical comparisons are reported for at least one key outcome	Figures 3, 4	Table 2	p.13, Regression, Tables 2, 3	Table 3	Table 2	Table 3	
11. The study provides both point measures and measures of variability for at least one key outcome	p.10, Findings	Table 2	Tables 2, 3	Table 4	p.225, Prelim effects	Table 5	
Total	9/11	7/11	8/11	7/11	7/11	6/11	

Information in each cell indicates the page number or section (Abstract, Methods, Results, Participants, Design, Statistics analysis, Outcomes, Comparison, Effect size, Tables, Figures, etc.) under which each of the 11 areas of quality assessment was reported by the study authors.

TAI, transfer assessment instrument.

Telephones and computers were the more commonly used technology for delivery of the intervention, and all required internet connection.^6,22,23,26,27,29^ Computer-based platform was used for automated interactive voice response system,^32^ videoconferencing,^24^ and web-based internet communications.^11,27^ One study required smartphones while another used a wearable remote wireless monitoring device.^31^ Three studies also required secondary technology equipment, including computers for online data tracking system,^22,24,26^ and one study provided participants with laptops, computers, and web-cams.^24^

In this review, one study addressed privacy concerns in protecting participants' personal information acquired through an online survey.^25^ All other studies did not report achieving data encryption or securing of personal information in adherence to Heath Insurance Portability and Accountability Act (HIPAA) guidelines.

#### Study results and outcomes data

Primary outcomes observed were as follows: all-cause mortality,^6^ pressure ulcer and depression,^32^ mood improvement with life satisfaction,^32^ costs and health outcomes with quality-adjusted life years gained,^23^ upper limb strength and function,^24^ depression symptoms severity,^25^ self-management in health techniques,^26^ health and psychosocial outcomes,^11^ 6-min walk or push test,^27^ changes in score for wheelchair transfer techniques,^28^ biomedical (weight, body mass index, barrier to activity), physical activities, nutritional status (scores in fat, fiber, and fruit/veggie),^29^ securing employment, and depression symptoms severity.^30^

Secondary outcomes were assessed in five studies: medical complications, depression, participation, QoL; hand (grasp and pinch forces) function; anxiety, pain perception, catastrophizing, and mindfulness; global ratings of services or resource use; and quality of primary care.^6,25,26,28,30^

Outcome measurements were primarily quantitative and demonstrated a positive impact. Among 43 total measurements tested across studies for statistical significance, 34 (79.1%) were significant for positive outcomes, whereas 8 (18.6%) yielded no effect but were significant. There was one negative impact by statistical testing,^23^ and one study reported no significant testing for outcome measures.^6^ Table 3 summarizes the primary outcomes and measurements.

Attrition rates across all studies ranged from 6.7% (2 out of 30)^6^ to 36% (9 out of 40 unaccounted for in final analysis).^30^ Only one study had all of their participants complete the study,^25^ and one study did not report any information on attrition.^23^ Three participants withdrew due to disinterest,^11^ and seven withdrew for unspecified reasons.^30^ Adverse events across studies were as follows: three deaths,^6,11^ seven hospitalizations for pressure ulcers,^6,29^ one participant started dialysis,^29^ and two participants had acute illnesses.^30^

## Discussion

Telemedicine concepts and application in clinical practice have demonstrated their feasibility over the past decade. However, teleSCI is yet to become widely implemented worldwide. The focused areas of investigation among the reviewed studies included community-based care after discharge from inpatient rehabilitation, wound care management, orthostatic hypotension, mental health, chronic pain, hand function, home physiotherapy, wheelchair transfer training, bodyweight management, promoting self-management in health, psychosocial support, and securing of employment for community-dwelling individuals with SCI.^6,11,22–32^

In our search, 13 teleSCI studies met inclusion criteria, which were finally included in this review. This is a significant contrast from a review of telemedicine studies in managing other conditions—asthma, chronic obstructive pulmonary disease, diabetes, heart failure, and hypertension—as demonstrated by Wootton that identified >1300 publications.^33^ And thus, the observation validates the reality of sparse data in the literature on teleSCI investigations.

Our review of selected studies was representative of the SCI population across characteristics such as gender, age, and type of injury. A review of global prevalence and incidence of traumatic SCI shows a high male-to-female ratio^34^ and is consistent with the majority of the studies reviewed. The average age of injury, at 42 years,^35^ is consistent with the central tendency of participant ages represented within the studies. The predominant injury type reported was incomplete SCI, consistent with epidemiological trends (66.3% of all SCI).^35^ All studies were published in English, with many having conducted in North America. However, given the absence of an accurate global prevalence estimate, establishing geographic representation is problematic^34,36,37^ and is indicative of the need for standardization of guidelines for global SCI reporting.^36,37^

The lack of standardized neurological levels of injury reporting was evident across studies. This deficit can potentially lead to challenges in clinical practice and in understanding current works and future needs in SCI research.^36,37^ However, in this review, only two studies appropriately reported neurological levels of injury using the standardized ASIA impairment scale classification.^6,11^ The majority of studies used generalized terms such a “tetraplegics” or “paraplegics.” Others merely reported types of injury, such as “complete” or “incomplete.” Reviewers must be aware of these inconsistencies as critical barriers to accuracy in the reporting of results. The consistency with standardized reporting is compulsory since it minimizes doubts and errors while enhancing clear communication between research scientists and clinicians within the academic community. By a committee of international experts, these limitations have been recognized and prompted to promote global standards of reporting in future SCI studies.^38^ One area of standardization suggested by the committee is shared data and minimum information standards^38,39^ to ensure highly improved results from systematic review studies.

This review's selected studies are all randomized trials, of which four are pilot or feasibility studies.^6,11,25,30^ Six studies reported details, following Consolidated Standards of Reporting Trials (CONSORT) guidelines, in recruitment, randomization, and ascertaining of allocation concealment.^6,22,26,27,28,30^ Two studies neglected to report any information on these processes.^23,32^ Among the reviewed studies, <50% (6 of 13) achieved blinding of participants,^6,22–26^ whereas some studies secured blinding of research staff involved in either the delivery of interventions (2 of 13)^11,24^ or assessing outcomes data (6 of 13).^6,11,23–25,29^ Thus, potential biases across studies appear to be associated with blinding and allocation concealment. Our quality assessment based on PEDro scale^20^ revealed that <40% of all studies achieved allocation concealment, and a mere 46% of studies incorporated blinding in their study designs (Table 4). Based on this rating scale assessment, it is plausible that 81.8% (9 of 13) of the reviewed studies are likely to be reliable.

We observed primarily positive outcome measures (79.1%) across studies. Similarly, most secondary outcome measures (38.5%) were positive and focused on psychosocial issues in the management of mood and depression.^25,26,32^ One area, which lacked the attention that it deserves among these individuals, is erectile dysfunction. The highly prevalent condition among SCI males resulting in sexual dysfunction and infertility issues severely impacts life quality.^40^ Among the reviewed studies, this area represents a knowledge gap, which indicates the need for further research in SCI sexual rehabilitation.^41^ In contrast, it is arguable that SCI men who are confronted by this issue may be more inclined to prefer personalized face-to-face attention in addressing this sensitive area in the rehabilitation process.

The studies used various methods to support SCI patients in the home and community through teleSCI interventions using various technologies. The common mode of intervention delivery included telephones and computers for videoconferencing, interactive voice response, and online data messaging systems. Web-based platforms, as expected, were widely used and still remain the conventional technology. This is consistent with the general trend in telemedicine practices for other medical conditions.^42,43^ However, judging from the consistency of traditional technologies used in earlier studies considered to date,^42,43^ not much has evolved to yield innovative or cutting edge technology in recent years, at least not in the extent to which it would have been considered applicable to drastically impact telerehabilitation practices. The void brought on by this lack of technology advancement—observed across studies of recent years—maintains use of conventional platforms as status quo.^42,43^

Although earlier innovations may have served as driving forces toward the significant development of telemedicine, technological performance and applicability may be one of the biggest challenges to implementing teleSCI across the globe. Secondary technologies were also required in many situations. These included webcams, smartphones, speakers, microphones, portable laptops, remote wireless monitoring systems, and wearable devices. The concurrent reality is that many elderly patients who require ongoing rehabilitation are unfamiliar with using smart devices, whereas individuals with limited function likely require assistance in operating technological devices. Reliable internet connections with adequate speed and the need for secondary equipment with knowledge in their operability are tangible challenges^44,45^ to stabilizing and ensuring the flawless delivery of interventions for these patients' optimal clinical care. These challenges are limiting factors and serve as catalysts in the future direction for teleSCI research and clinical practice.

The advent of smart technology has given birth to a myriad of lifestyle conveniences. It has also served to be pivotal in the delivery of health care through telemedicine. Ironically, it has also resulted in costly losses for many victims falling akin to hacking schemes and identity theft due to personal information security deficiencies. Data security and maintenance of the privacy of personal information are critical issues in clinical practice. Those involved in handling such sensitive information must abide by HIPAA compliance standards and other security regulations.^46–48^ In contrast, most studies in our review failed to address privacy issues and data protection. Patients' records should be kept highly secured, intact, and protected from third-party theft or access without consent.^49^ They should be accessible solely to authorized individuals only when needed, strictly for purposes of health care delivery or medicolegal situations. Security systems such as the Intrusion Detection System—automatically detect malicious activities and report to security service providers^50^—or similar security systems should be mandated to handle data acquisition of private and personal information in research activities. The prevention of unintended leakage of and securing personal data remain a critical challenge in teleSCI. Hall and McGraw validate this by asserting in the title of their article: “for telemedicine to succeed, privacy and security risks must be identified and addressed.”^48^

The burdensome question of cost-effectiveness and benefits to both patients and providers alike will likely impact decisions regarding the implementation of teleSCI.^51^ Telemedicine practices in other specialties have shown reduced costs compared with traditional face-to-face visits. It yields an overall decrease in treatment costs and transportation costs by minimizing the frequency of in-person clinic appointments.^50^ Furthermore, it reduces time out of office costs for employees and employers by eradicating the need to take time off from work. Arora et al.—whose study failed to meet inclusion criteria and thus excluded in this review—validated the cost–benefits of teleSCI intervention using technology among participants with pressure injuries.^52^ Since SCI rehabilitation is an ongoing and expensive process, the use of teleSCI may decrease financial burdens on patients and the health care system. However, further studies are needed to draw meaningful conclusions in areas of cost-effectiveness for SCI management.

### Limitations

Although the studies in this systematic review addressed different aspects of SCI management using conventional technology platforms, the study's inclusion criteria served to be a limitation by failing to capture a sizable compilation of journal articles. Consequently, the included studies' heterogeneity made comparison difficult across selected studies, particularly for quantitative analysis of outcome measures.

## Conclusion

The majority of studies in this review demonstrated significant positive outcomes to validate current teleSCI practices' clinical effectiveness using conventional technology. The qualitative synthesis results further expand our understanding of teleSCI's impact and its demonstrated potential for improving SCI individuals' lives. However, the development of new data generated by ongoing research efforts to promote the “buy-in” toward widespread teleSCI implementation is warranted.

## Abbreviations Used

ARAT: Action Research Arm Test
CI: confidence interval
CP: cerebral palsy
ePACT: electronic personal admin of cognitive therapy
ES: effect size
ET: exercise therapy
HIPAA: Heath Insurance Portability and Accountability Act
JSES: Job Procurement Self-Efficacy Scale
MPT: Min Push Test
MS: multiple sclerosis
MWT: Min Walk Test
OR: odds ratio
PAM: Patient Activation Measure
PEDro: Physiotherapy Evidence Database
QALY: quality-adjusted life years
QoL: quality of life
RCT: randomized controlled or clinical trial
SCI: spinal cord injury
SCI/D: spinal cord injury or dysfunction
teleSCI: spinal cord injury telerehabilitation

## Appendix A1

**Table:** Appendix A1. Abbreviations

WHO-QOL-BREF	WHO-QoL-Brief Instrument Form
HR-QoL	health related-QoL
PWI	Personal Well-being Index
SCLWL	Spinal Cord Lesion Emotional Well-being
PHQ-9	Patient Health Questionnaire-9
QALY	quality-adjusted life years
PUSH	Pressure Ulcer Scale for Healing
NRS	numerical rating scale
PCS	Pain Catastrophizing Scale
CES-D	CES Depression Scale
DASS-21	Depression Anxiety and Stress Scale
BDI-II	Beck Depression Inventory-II
HADS	Hospital Anxiety and Depression Scale
CHART-SF	Craig Handicap Assessment and Reporting Technique Short Form
PADS	Physical Activity and Disability Survey
SCI-SC	SCI Secondary Conditions Scale
SCIM-III	Spinal Cord Independence Measure
COPM	Canadian Occupational Performance Measure
WHODAS	WHO Disability Assessment Schedule
CHART-SF	Craig Handicap Assessment and Reporting Technique Short Form
FFMQ	Five Facet Mindfulness Questionnaire
PAM	Patient Activation Measure
PACIC	Patient Assessment of Chronic Illness Care
ASIS	Adolescent Self-Management and Independence Scale
CHIEF-SF	Craig Hospital Inventory of Environmental Factors-Short Form
ARAT	Action Research Arm Test
RAHFT	ReJoyce Automated Hand Function Test
TAI	Transfer Assessment Instrument
JSES	Job Procurement Self-Efficacy Scale
LOT-r	Life Orientation Test-Revised.
